# Steady state visual evoked potentials in schizophrenia: A review

**DOI:** 10.3389/fnins.2022.988077

**Published:** 2022-10-28

**Authors:** Alexander Schielke, Bart Krekelberg

**Affiliations:** Center for Molecular and Behavioral Neuroscience, Rutgers University, Newark, NJ, United States

**Keywords:** steady state, visual, evoked potentials, schizophrenia, EEG, SSVEP

## Abstract

Over the past decades, researchers have explored altered rhythmic responses to visual stimulation in people with schizophrenia using steady state visual evoked potentials (SSVEPs). Here we systematically review studies performed between 1954 and 2021, as identified on PubMed. We included studies if they included people with schizophrenia, a control group, reported SSVEPs as their primary outcome, and used quantitative analyses in the frequency domain. We excluded studies that used SSVEPs to primarily quantify cognitive processes (e.g., attention). Fifteen studies met these criteria. These studies reported decreased SSVEPs across a range of frequencies and electrode locations in people living with schizophrenia compared to controls; none reported increases. Null results, however, were common. Given the typically modest number of subjects in these studies, this is consistent with a moderate effect size. It is notable that most studies targeted frequencies that fall within the alpha and beta band, and investigations of frequencies in the gamma band have been rare. We group test frequencies in frequency bands and summarize the results in topographic plots. From the wide range of approaches in these studies, we distill suggested experimental designs and analysis choices for future experiments. This will increase the value of SSVEP studies, improve our understanding of the mechanisms that result in altered rhythmic responses to visual stimulation in schizophrenia, and potentially further the development of diagnostic tools.

## Introduction

Steady state visually evoked potentials (SSVEPs) emerge during the presentation of visual stimuli whose parameters (e.g., luminance, contrast) are modulated rhythmically at a set frequency. EEG, with its high temporal resolution, is often used to measure SSVEPs and, depending on the frequencies present in the visual input, often reveals strong, rhythmic neuronal responses. These responses provide insight into the processing performed by the underlying neural circuitry (for review, see Norcia et al., 2015).

The spectral signatures related to the processing of visual information are altered in people living with schizophrenia (Sz) (Green et al., 2003; Spencer et al., 2003, 2004, 2008; Wynn et al., 2005; Uhlhaas et al., 2006; Grützner et al., 2013). Notably, these changes are accompanied by changes in visual processing (Dakin et al., 2005; Kantrowitz et al., 2009; Horton and Silverstein, 2011; Keane et al., 2014, 2022; Schallmo et al., 2015), and visual aberrations are related, even prodromally, to the severity of clinical symptoms (Phillipson and Harris, 1985; Uhlhaas and Mishara, 2007; Keane et al., 2018).

Based on these considerations, several studies have used SSVEPs to gain a better understanding of rhythmic neuronal activity in Sz. Evaluating the overall evidence in favor of alterations in Sz, however, is complicated by the wide range of experimental approaches. For instance, studies differ in terms of the frequencies they probe, the placement of EEG electrodes, or the analysis methods they apply. The primary goal of this review is to summarize the aggregated evidence in a frequency-band and electrode-location specific manner. In addition, our goal is to provide an overview of the commonly used stimulus and analysis parameters and, from these, suggest best-practices for future studies.

## Methods

### Study selection

**Figure 1:**
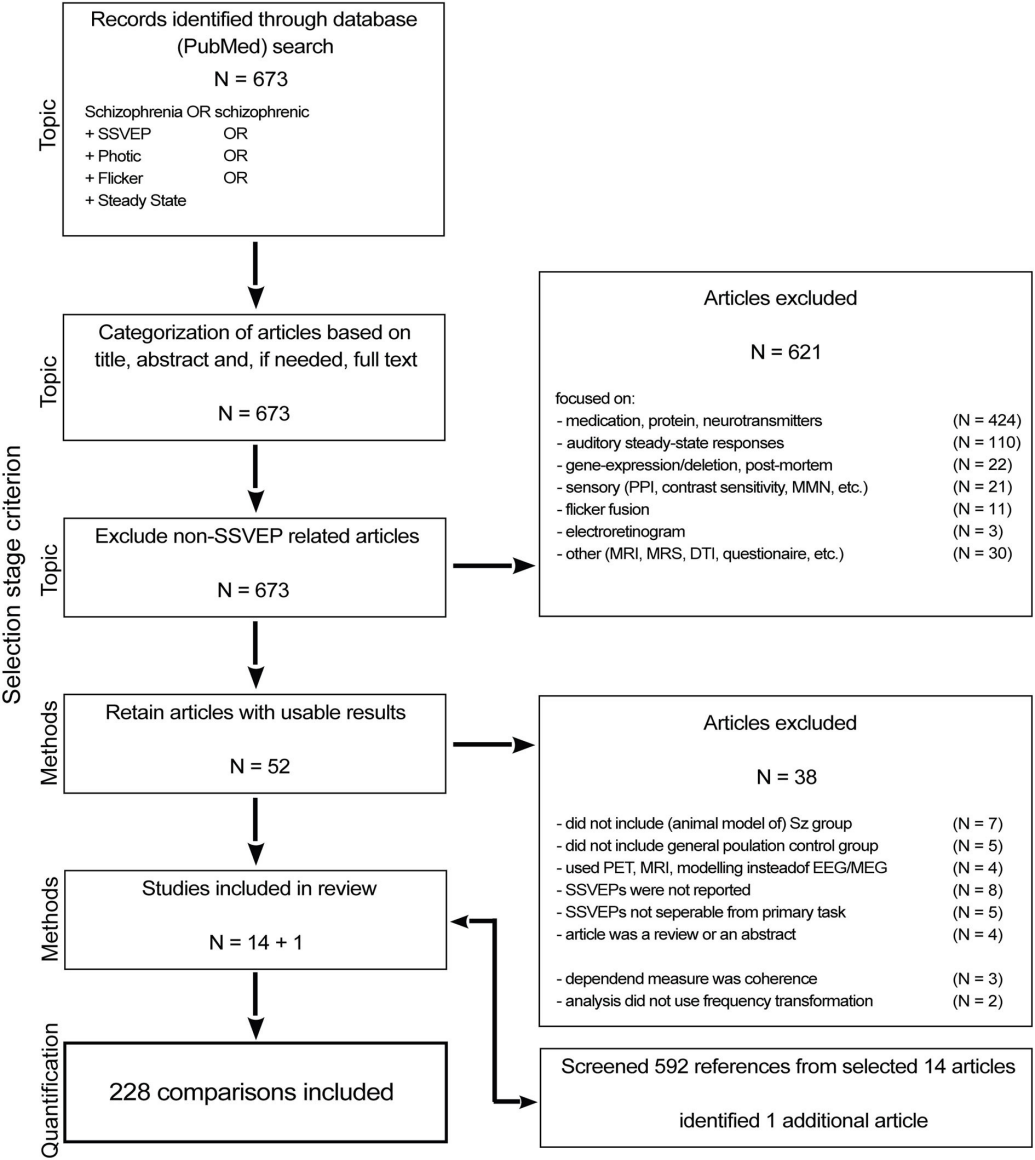
Flow chart of the article selection process.

We initially identified 673 research articles by searching the PubMed database (last accessed on August 1st, 2021) for articles between 1954 and 2021 that contain either the term “schizophrenia” or “schizophrenic” in the title or abstract in addition to either one of the following: “ssvep” (steady state visual evoked potential), “photic,” “flicker,” “steady state,” or “pdr” (photic driving response) (Figure 1). From the resulting 673 articles, we first removed articles based on their overall topic [i.e., we removed those that were not investigating SSVEPs (621)]. Four hundred and twenty-four of those articles focused on medication effects or on the role of proteins and neurotransmitters. One hundred and ten articles measured the auditory steady state response and 22 of those were animal studies. Another 22 articles were gene expression/deletion (18) as well as post-mortem studies (4). Other measures of sensory integrity, including topics such as pre-pulse inhibition, smooth pursuit eye movements, oddball/mismatch negativity, steady state tactile evoked responses and auditory evoked responses were the content of 21 articles. Eleven articles investigated flicker fusion and 3 measured the electroretinogram (1 animal study). Lastly, 31 of the 621 reports that we removed at this stage were on topics such as MRI, MRS and DTI or were mainly model-based or concerned with non-medical treatment.

After the topic-based selection, 52 articles remained; these were subjected to selection based on the experimental design. We included only studies with both a group of people diagnosed with schizophrenia and a control group of the general population (11 excluded). We excluded 4 articles that measured the SSVEP using PET, MRI or modeling instead of with EEG or MEG. Another 8 studies used SSVEP stimuli in their experimental manipulation but did not include measures of those SSVEPs in their report. Furthermore, because task demands could affect intrinsic oscillatory activity separate from the visual drive, we excluded studies in which the task demands went beyond steady fixation (5).

The third selection step evaluated the (quantification of) the dependent variable, we excluded 3 studies measuring coherence (as opposed to power) (Wada et al., 1998a,b; Riečanskỳ et al., 2010), and 2 articles (Jibiki et al., 1991; Wada et al., 1995) used a qualitative measure instead of power to quantify the SSVEP strength. To decrease the likelihood of missing relevant articles we went through the reference lists of the remaining 14 articles and identified 1 additional article (Butler et al., 2005) that fit all criteria. As a result, we included 15 articles in this systematic review.

### Summarized results

A formal meta-analysis using effect sizes was not possible since few publications contained the necessary information to calculate effect size. As an alternative, we summarized the results by tabulating (for each recording site/electrode and each frequency band; see below) whether a study found evidence for either a decrease or increase in power at a significance level of 0.05. We did not blindly include all reported comparisons from each study. For instance, we excluded comparisons of SSVEP at frequencies other than the harmonics (Goldstein et al., 2015), comparisons of activity before and after the presentation of SSVEP stimuli, or comparisons between groups for harmonics that failed to evoke a significant SSVEP in either group (Butler et al., 2001; Kim et al., 2005; Calderone et al., 2013). Across the 15 publications, our selections resulted in 228 relevant *post-hoc* tests with the parameters shown in **Table 3**. The summarized results show the fraction of these tests that resulted in decreases, increases, or non-significant differences between the Sz and control group.

Data were aggregated and visualized (proportions of positive, negative, and non-significant changes within frequency bands and across recording sites) with MATLAB (RRID:SCR_001622).

### Electrode sites

Most studies used the 10–20 system to identify electrode locations; we adopted this same convention to aggregate electrode-specific findings across studies. If a study reported an effect at electrode clusters instead of a specific electrode site, we assigned the result to multiple electrodes according to the 10–20 system, but still counted it as one test. For example, if a study used statistical parametric mapping and reported an effect at a temporal cluster, then we assigned the result to the electrodes T5, T4, C3, C4, T5 and T6. Other clusters were mapped as follows: frontal/anterior: (Fz, Fp1, Fp2, F3, and F4), central: (Fz, Cz, Pz, C3, and C4), occipital/parietal/occipital-parietal: (Pz, Oz, P3, P4, O1, and O2). If authors used the average of all their recording sites, then we assigned the result to the intersection of their recording sites with the following set of electrode sites (Fp1, Fp2, F7, F3, Fz, F4, F8, T3, C3, Cz, C4, T4, T5, P3, Pz, P4, T6, O1, Oz, and O2).

### Frequency bands

The definitions of the five frequency bands (delta, theta, alpha, beta, and gamma) differed somewhat across studies. To aggregate across studies, we followed the definition used in each study. For example if the study assigned a frequency to the alpha band, we analyzed it as part of the alpha band, regardless of the frequency. If the authors did not use band labels, we assigned frequencies to bands as follows: delta (0, 4), theta [4, 8), alpha [8, 13), beta [13, 32), gamma [32, 120). With these definitions 12 (5.26%) tests targeted the delta band, 58 tests (25.44%) the theta band, 103 tests (45.18%) the alpha band, 51 tests (22.37%) the beta band and 4 tests (1.75%) the gamma band. We use these bands because it is common practice in these studies and allowed us to combine results across studies in this review. The use of these bands to describe the visually driven responses does not imply a link with intrinsic oscillations in these bands (see Section Discussion).

### Effect size estimates

We used the fraction of significant results across studies to estimate the underlying true effect size, using Monte Carlo simulations separately for each band and electrode. Using random draws from a normal distribution, we created 1,000 simulated data sets with the number of subjects and the number of tests matching the data set (i.e., **Table 3**) but with an assumed effect size (Cohen's D) that varied from 0 to 2 in steps of 0.05. For each simulated data set, we determined the fraction of significant tests (based on an equal variance two-sample *t*-test at a significance level of 0.05) and then determined the average fraction of significant tests across the 1,000 simulated data sets. We defined the estimated effect size as the smallest simulated effect size for which the fraction of significant tests in the simulation matched or exceeded the fraction of significant tests in the data.

## Results

We identified and reviewed 15 studies investigating SSVEP in people living with schizophrenia. We first present an overview of salient methodological differences across studies and, based on these, our recommendations for future work. Second, we summarize the outcomes of these studies.

### Research approaches

Even across this relatively small number of studies, the variation in the approach is considerable. Tables 1–3 summarize key demographic information and stimulus and analysis choices. We discuss a subset of salient differences and their consequences for the interpretation of the data.

**Table 1:** Demographic and trial information.

**References**	**Groups**	**Participants (female)**	**Age**	**Trials per condition**	**Segments per trial**
Rice et al. (1989)	Sz	8 (0)	26 (*SD* = 4.28)	8	1
	Controls	11 (0)	24.3 (*SD* = 3.93)		
Jin et al. (1990)	Sz	8 (0)	23.9	8	1
	Controls	11 (0)	24.3		
Wada et al. (1994)	Sz	14 (7)	23.3 (*SD* = 4.15)	1	3
	Controls	20 (10)	23.1 (*SD* = 2.51)		
Jin et al. (1995)	Sz	17 (3)	31.8 (*SD* = 8.3)	8–10	4
	Controls	15 (2)	29.4 (*SD* = 6.2)		
Jin et al. (1997)	Sz	38 (11)	31.4 (*SD* = 7.7)	8	1
	Controls	24 (14)	30.8 (*SD* = 9.7)		
Jin et al. (2000)	Sz	27 (6)	31.6 (*SD* = 11.2)	1	30
	Controls	25 (12)	26.2 (*SD* = 6.6)		
Butler et al. (2001)	Sz	24 (0)	49.2 (*SD* = 11.5)	10	6–7 (1 per condition)
	Controls	22 (0)	50.5 (*SD* = 9.1)		
Kikuchi et al. (2003)	Sz	18 (6)	22.8 (*SD* = 4)	6	10
	Controls	18 (6)	25.9 (*SD* = 6.7)		
Clementz et al. (2004)	Sz	12 (5)	30.4 (range 18–54)	32	14
	Controls	12 (5)	30.6 (range 19–50)		
Butler et al. (2005)	Sz	32 (7)	37.1 (SEM = 1.7)	10	7 (1 per condition)
	Controls	20 (8)	26.2 (SEM = 2.2)		
Kim et al. (2005)	Sz	26 (5)	38.7 (*SD* = 10.5)	6	1
	Controls	22 (12)	39.2 (*SD* = 10.7)		
Krishnan et al. (2005)	sz	18 (7)	39.5 (*SD* = 7.6)	1	1
	Controls	33 (15)	36 (*SD* = 9.9)		
Ethridge et al. (2011)	Sz	12 (4)	36.4	66	1
	Controls	12 (6)	35.2		
Calderone et al. (2013)	Sz	15 (3)	40.4 (*SD* = 9.9)	10	7 (1 per condition)
	Controls	15 (2)	36.8 (*SD* = 10.01)		
Goldstein et al. (2015)	sz	13 (5)	33.2 (*SD* = 10.7)	1	30
	Controls	13 (3)	38.2 (*SD* = 11.2)		

The number of trials is stated per condition, and the number of segments is defined per trial (e.g., Goldstein et al. used a single trial (of 120 s; see Table 2) per condition and extracted 30 segments (of 4 s) from each trial for their analysis.).

**Table 2:** Recording and experimental parameters.

**References**	**Filter (Hz)**	**Device/Stimulus**	**Stimulus Duration (Segment Duration)**	**Stimulus intensity**	**Frequency resolution**	**Maximum impedance**
Rice et al. (1989)	0.3–35	Goggles 60 red LEDs	10 s (10 s)	36 millicandela per goggle	0.1 Hz	N.A.
Jin et al. (1990)	0.1–35	Goggles	10 s (10 s)	N.A.	0.1 Hz	N.A.
Wada et al. (1994)	0.3–60	Strobe	30 s (5 s)	5,023 cd/m^2^	0.2 Hz	< 5 kOhm
Jin et al. (1995)	0.1–70	Goggles	10 s (2.5 s)	N.A.	0.4 Hz	N.A.
Jin et al. (1997)	0.1–70	Goggles	10 s (10 s)	N.A.	0.1 Hz	N.A.
Jin et al. (2000)	0.1–70	Strobe	120 s (4 s)	1.28 J	0.25 Hz	< 5 kOhm
Butler et al. (2001)	0.1–100	Checker-board sinusoidal black/white, 2 × 2, 4 × 4, 8 × 8, 16 × 16, 32 × 32, 64 × 64, squares red/yellow 32 × 32 squares	7 s (1 s)	100 cd/m^2^	1 Hz	N.A.
				8, 16, and 32% depth of modulation		
				60 cd/m^2^ 40, 80, and 100% depth of modulation		
Kikuchi et al. (2003)	0.1–60	Strobe	10 s (1 s)	5,023 cd/m^2^	1 Hz	< 5 kOhm
Clementz et al. (2004)	N.A.	Checker-board square wave 8 × 8 red squares	2, 4, 6 s (312.5 s)	6.3 cd/m^2^	1.28 Hz	N.A.
Butler et al. (2005)	0.1–100	Checker-board sinusoidal black/white	7 s (1 s)	100 cd/m^2^ mean luminance 1, 2, 4, 8, 16, and 32% depth of modulation	1 Hz	N.A.
Kim et al. (2005)	0.1–100	Dartboard sinusoidal contrast reversal	60 s (60 s)	100 cd/m^2^ mean luminance ±32% contrast modulation	0.017 Hz	N.A.
Krishnan et al. (2005)	0.1–100	LED sinusoidal	120 s (100 s)	300–800 cd/m^2^	0.01 Hz	< 10 kOhm
Ethridge et al. (2011)	0.5–75	Checker-board 2 (left, right) 8 × 8 red/black	2 s (0.5 s)	N.A.	2 Hz	< 50 kOhm
Calderone et al. (2013)	0.5–100	Checker-board sinusoidal black/white 16 × 16 squares	7 s (1 s)	50 cd/m^2^ 4, 8, 16, and 32% depth of modulation	1 Hz	N.A.
Goldstein et al. (2015)	0.5–50	LED sinusoidal	120 s (4 s)	100 cd/m^2^ mean luminance ±32% contrast modulation	0.25 Hz	N.A.

Stimulus intensities refer to the intensities that were included in our analysis (see Section Methods).

**Table 3:** Stimulus and analysis parameters.

**References**	**Analyzed electrode locations**	**Electrodes clustered**	**Stimulus frequencies**	**Analysis frequencies**	**Frequency bands investigated**	**Eyes**
Rice et al. (1989)	Fz and Pz	No	2.4	2.2–2.6, 4.6–5, 7–7.4, 9.4–9.8, and 11.8–12.2	Delta, Theta, Alpha, and Beta	Closed
			4.5	4.3–4.7, 8.8–9.2, 13.3–13.7, 17.8–18.2, and 22.3–22.7		
			8.3	8.1–8.5, 16.4–16.8, 24.7–25.1, and 33–33.4		
Jin et al. (1990)	Fz and Pz	No	2.38	2.38, 4.76, 7.14, 9.53, and 11.9	Delta, Theta, Alpha, Beta	Closed
			4.54	4.54, 9.08, 13.62, 18.16, and 22.7		
			8.33	8.33, 16.66, and 25		
Wada et al. (1994)	Fp1, Fp2, F3, F4, C3, C4, P3, P4, O1, O2, F7, F8, Fz, Pz, T5, and T6	No	10	4.8–5.2, 9.8–10.2, and 19.8–20.2	Theta, Alpha, and Beta	Closed
Jin et al. (1995)	Fp1, Fp2, F3, F4, C3, C4, P3, P4, O1, O2, F7, F8, Fz, Pz, T5, T6, T3, and T4	Frontal, Central, Temporal, and Parietal/Occipital	3.125	2.8–3.2, 6–6.4, 9.2–9.6, and 12.4–12.8	Delta, Theta, and Alpha	Closed
			6.25	6–6.4 and 12.4–12.8		
			12.5	12.4–12.8		
Jin et al. (1997)	Fz, Pz, and Oz	No	2.4, 4.5, and 8.3	7.2, 8.3, 9, and 9.6	Delta, Theta, and Alpha	Closed
Jin et al. (2000)	Fp1, Fp2, F3, F4, C3, C4, P3, P4, O1, O2, F7, F8, Fz, Pz, T5, T6, T3, and T4	SPM—Frontal, Temporal, and Parietal/Occipital	1	7.75–8.25, 8.75–9.25, 9.75–10.25, 10.75–11.25, 11.75–12.25, and 12.75–13.25	Alpha	Closed
Butler et al. (2001)	Oz, “parietal site”	No	6	6	Theta and Alpha	Open
			12	12		
Kikuchi et al. (2003)	F3, F4, C3, C4, P3, P4, O1, and O2	No	10	9–11	Alpha	Closed
Clementz et al. (2004)	Po7, Po8 and each ones' 5 nearest neighbors	Average	6.4	6.4	Theta	Open
Butler et al. (2005)	Occipital midline	No	12	12	Alpha	Open
Kim et al. (2005)	Oz	No	4	4, 8	Theta and Alpha	Open
Krishnan et al. (2005)	Oz, Fz	No	4, 8, 17, 20, 23, 30, and 40	3.75–4.25, 7.75–8.25, 16.75–17.25, 19.75–20.25, 22.75–23.25, 29.75–30.25, and 39.75–40.25	Theta, Alpha, Beta, and Gamma	Closed
Ethridge et al. (2011)	29 posterior electrodes (geodesic net)	Average	12.5	12.5	Alpha	Open
Calderone et al. (2013)	Oz	No	12.5	12.5	Alpha	Open
Goldstein et al. (2015)	256 geodesic electrode net	Frontal and occipital	10	8–12	Alpha	Closed

This table shows the wide range of approaches followed across the reviewed studies. Stimulus and analysis frequencies refer to the frequencies that were included in our analysis (Methods).

#### Equipment

Early studies generally recorded from fewer electrodes, filtered with lower cut-off frequencies (e.g., < 35 Hz), and required lower electrode impedances (Wada et al., 1994; Jin et al., 2000; Kikuchi et al., 2003). Most current EEG equipment can sample large numbers of electrodes at a high sampling rate and maintain good signal quality even with impedances up to 50 kOhm (Ethridge et al., 2011). This enables whole-brain studies, and provides a window into the gamma band where signals are generally smaller.

Stimulus presentation devices included light emitting diodes (LEDs, sometimes mounted inside goggles), stroboscopic lamps, and computer monitors. LEDs and stroboscopic lamps have two advantages. First, they can generate very high intensities. This can be advantageous if the magnitude of differences increases with stimulus intensity (Butler et al., 2001, 2005; Calderone et al., 2013) or to generate a high luminance (e.g., 5,023 cd/m^2^) that generates SSVEPs even when the subjects' eyes are closed. The other advantage is that their intensity can be modulated continuously, which allows the experimenter to generate any desired temporal pattern. Computer monitors are much more limited in the intensities and temporal patterns they can produce. Their refresh rate (typically below 120 Hz) limits both the maximum attainable frequency (half the refresh rate; 60 Hz) and the frequencies that can be generated (integer divisions of the refresh rate; 40 Hz, 30 Hz, 24 Hz, 20 Hz, etc.). The primary advantage of computer monitors is that they can present spatially complex patterns (Butler et al., 2001, 2005; Clementz et al., 2004; Kim et al., 2005; Calderone et al., 2013) and are sometimes indispensable for other aspects of the experiment (e.g., providing instructions, or a central fixation point). Furthermore, the ability of computer monitors to present spatially complex stimuli allows experimenters to alter contrast in a spatial pattern in addition to modulating luminance (Butler et al., 2001, 2005; Calderone et al., 2013). Over the past years, projectors with presentation frequencies of 1,400 Hz and above have become available; they are an attractive alternative to present complex spatial patterns at fast presentation rates.

#### Eyes open or closed

In 9 out of 15 studies, subjects were instructed to keep their eyes closed during visual stimulation. Using high-luminance devices, this nevertheless generates measurable SSVEPs. The main advantage of this approach is that stimuli can be presented for a long time (10–120 s) and that eye-blink or rapid eye-movement artifacts in the EEG recordings are minimized. Eyes-closed recordings are also common in studies of resting state activity, where they generate more consistent findings across studies (Newson and Thiagarajan, 2019). On the other hand, closing the eyes typically results in drowsiness and increased power in the alpha band; this potentially confounds an interpretation of group differences and impedes a direct comparison to the outcomes of eyes-open studies. Moreover, one of the primary functions of the visual system is, of course, to process spatial contrast. Therefore, if one wants to understand how schizophrenia affects the mechanisms of visual processing, an experiment with the eyes open seems a better choice. Finally, eyes-open experiments are also easier to translate for use in animal models.

The downside of eyes-open experiments is that subjects will make blinks and move their eyes, which results in contamination of the EEG signals. Blinks are often separated by less than 10 s (Doughty, 2001) and natural fixations typically last less than 300 ms (Einhäuser et al., 2020). Hence, even in short trials contamination is likely. This problem is exacerbated by the finding that eye movements are altered in Sz (Miura et al., 2014; Morita et al., 2020), thereby introducing not only variability but a potentially confounding factor.

Some studies (e.g., Kim et al., 2005) have mitigated these factors by visually monitoring the subject and the EEG recording for unstable gaze and blinking and rejecting trials with excessive artifacts. Another strategy is the use of artifact correction techniques. For instance, two eyes-open studies (Clementz et al., 2004; Ethridge et al., 2011) used BESA for correction of blink artifacts (Berg and Scherg, 1994). A third option is to use shorter trials, which reduces the number of eye movements and blinks (see below). In all cases, the use of an infrared eye-tracker is advisable as an additional source of information about the status of the eyes.

#### Temporal modulation of the stimulus

In a linear system, a sinusoidally modulated input will generate a sinusoidally modulated output with the same frequency. The ratio of the output and input amplitude is a frequency-dependent measure of the linear system's gain or transfer function. Deviations from linearity result in output modulation at integer multiples of the input frequency (higher harmonics). Hence by presenting sinusoidal luminance variations at different frequencies one can -in principle- determine both the linear and nonlinear components of the luminance response of the brain. The systematic mapping of a range of input frequencies using sinusoidal inputs, however, is time consuming and constrained by the limitations of the stimulus presentation device (above).

Most (8) studies used non-sinusoidal temporal modulation. The sharp (square) transitions result in an input pattern that has power not only at the fundamental frequency but also at higher harmonics (integer multiples of the fundamental). Hence, most studies did not study the response at a single frequency, but at a mixture of frequencies. An extreme example of this is the study of Jin et al. (2000) who used a stroboscopic lamp to present a brief pulse of 5 ms at a rate of 1 Hz. This generates power at 1 Hz but also almost equal power at 2 Hz, 3 Hz, 4 Hz etc. This is an elegant solution that probes a wide range of frequencies in a single trial. In a linear system, the responses at the corresponding frequencies would quantify the frequency-dependent gain of the brain. However, the brain is not a linear system, which makes these results more difficult to interpret. For instance, the response at 10 Hz could result from the (linear) response to the 10 Hz power of the stimulus, or from a nonlinear response (the 10th harmonic) of the 1 Hz component, the 5th harmonic of the 2 Hz component, the 2nd harmonic of the 5 Hz component, or some sum of those responses and their interactions. For a diagnostic marker of a disease this may not be important, but if the goal is to understand which aspect of the underlying processing is different between groups, and how this may be related to changes in the neural circuitry, then this potential mixing of contributing components is undesirable.

For a more traditional sinusoidal modulation (7 out of 15 studies), the problem is less severe; such a pattern generates most power at the fundamental frequency, and the power of the response at the fundamental can be interpreted as the linear response of the system. The response at the second harmonic is a combination of the linear response to the second harmonic in the input, plus a nonlinear response to the fundamental. For sinusoidal modulation, these components can be disentangled in the analysis, at least for the lower order harmonics.

To summarize, the better the stimulus waveform approximates a sinusoid the more confident one can be in the interpretation of responses beyond the 1st harmonic and the information they provide about nonlinear processing in the brain. This helps gain mechanistic insight into which aspects of visual processing differ across groups. Kim et al. (2005), for instance, used this to argue that Sz specifically affects the nonlinear processing attributable to lateral connections in visual cortex.

#### Stimulus/trial duration

The use of long (≥10 s) trials is appealing and common (Rice et al., 1989; Jin et al., 1990, 1995, 1997, 2000; Wada et al., 1994; Kikuchi et al., 2003; Krishnan et al., 2005; Goldstein et al., 2015), as it poses minimal requirements on the subject. Moreover, long trials provide better frequency resolution and give access to power at low frequencies. Short trials, on the other hand, reduce the number of eye blink or eye movement artifacts (especially if the subject is instructed to blink in the intertrial interval), and they allow the experimenter to discard entire trials with artifacts rather than subtract artifacts from longer recordings. In addition, short trials can be preceded by baseline periods that allow the experimenter to assess spontaneous (non-stimulus driven) neural activity and correct for slow signal drift. Finally, using multiple short trials allows one to investigate intertrial coherence; the reliability with which each identical stimulus generates an identical response (see Section Discussion).

#### Analysis

Although the details vary (and are not always fully specified), the typical approach in published studies has been to first average the EEG signal across trials (sometimes excluding the initial 500 ms as a non-stationary onset period). The goal of averaging over trials is, of course, to reduce noise in the estimate, but it also removes response components that are not time-locked to the stimulus (e.g., induced power; see Discussion). Compared to the recommended number of trials in a typical ERP study (Thigpen et al., 2017), the number of trials in these SSVEP studies is low. Even though more trials could improve robustness, SSVEP analysis is possible with relatively few trials, because it focuses on a small range of the frequency spectrum, which has most of the signal, but only a small part of the noise (Regan, 1989; see Norcia et al., 2015 for discussion).

The next step of the analysis is to compute the power spectrum of the average response, and then average the power in a range of bins near the stimulus frequency. This average also appears to be to motivated by the goal to obtain a more robust estimate of the SSVEP, especially when the stimulus frequency falls between frequency bins. However, this average mixes stimulus driven responses with non stimulus-driven, intrinsic responses (at surrounding frequencies). Importantly, non-stimulus driven responses can increase or decrease as a result of rhythmic visual input and the relation between these changes and the frequency of the visual stimulus is not fully understood (Mast and Victor, 1991). Given these complexities, averaging over multiple frequency bins is not recommended as it could dilute the stimulus-driven response.

The studies we included in this review varied regarding how many frequency bins around the stimulus frequency authors averaged to estimate the strength of the SSVEPs and whether those analysis windows were centered around the stimulus frequency (Table 3). Six studies quantified SSVEP strength the recommended way, by selecting the single frequency bin at which the stimulus was presented (or its harmonics) (Jin et al., 1997; Butler et al., 2001, 2005; Clementz et al., 2004; Ethridge et al., 2011; Calderone et al., 2013). In five cases, the authors defined SSVEP strength as the average over multiple frequency bins, with the central bin matched to the stimulus frequency (or its harmonics) (Rice et al., 1989; Wada et al., 1994; Jin et al., 2000; Kikuchi et al., 2003; Goldstein et al., 2015). Jin et al. (1990) used 3.38, 4.54, and 8.33 Hz as presentation frequencies and report that they quantified SSVEPs at precisely those frequencies as well as their respective harmonics. However, given that they had a frequency resolution of 0.1 Hz it is not clear how they were able to extract frequency responses that were restricted to the precise stimulus frequency. Jin et al. (1995) averaged the 2 frequency bins that were nearest to each stimulus' frequency and their respective harmonics (i.e., for a 3.125 Hz stimulus frequency they used a 2.8–3.2 Hz analysis window). Lastly, Krishnan et al. (2005) cast a net of ±0.25 Hz around each stimulus' frequency and quantified SSVEP strength as the single frequency bin with the highest amplitude within that window.

It is important to emphasize that there is no need to average multiple frequency bins since SSVEPs have very narrow responses in the frequency domain (Meigen and Bach, 2000; Norcia et al., 2015). Bach and Meigen (1999) explain that signal dilution can be addressed in SSVEP paradigms by using a stimulus duration and analysis window that contains an integer number of cycles. With this simple change in the design, a simple, non-windowed, Fourier transform will capture the complete SSVEP in a single bin centered on the stimulus frequency (or one of its harmonics). An additional advantage of such a design is that long stimulus durations (often used to increase frequency resolution) are not required.

Using a stimulus with an integer number of cycles does not remove stimulus independent activity from the frequency bin of interest. To correct for the contribution of those sources Meigen and Bach (2000) suggest computing a signal-to-noise ratio (SNR) defined as the ratio of the magnitude at the stimulus frequency (stimulus: e.g., 10 Hz for a 10 Hz stimulus) relative to the magnitude in the frequency bins around the stimulus frequency (noise: e.g., 9 and 11 Hz). However, dividing SSVEPs by neighboring frequency bins poses some of the same concerns as averaging over multiple frequency bins (Mast and Victor, 1991). Others proposed using Hotelling T-squared (T^2^) or a related refinement called T_circ_^2^ (Victor and Mast, 1991) to estimate SNR. Of these three measures of SNR, only T${}_{\text{circ}}^{2}$ was used (Butler et al., 2001, 2005; Calderone et al., 2013) across the 15 studies selected for this review. All three methods have the advantage that they not only account for noise, but they also scale the response and therefore allow more direct comparison across (groups of) participants. However, caution is necessary regarding interpreting results when a dependent measure such as signal is scaled to another independent measure such as noise or pre-stimulus (baseline) activity. For instance, Ethridge et al. (2011) reported that, compared to controls, people with Sz had increased baseline activity and pointed out that using the ratio of stimulus driven activity to baseline activity can affect conclusions about group differences. If these findings are confirmed, these ratio measures could serve as useful biomarkers for Sz. However, because the mechanistic interpretation of an increase in noise/spontaneous activity is quite different from a decrease in stimulus driven activity (but could result in the same SNR), future studies should report both signal and noise changes, not only their ratio.

A final note on the statistical analysis in SSVEP studies is that analyzing multiple harmonics of multiple stimulus frequencies, at multiple electrodes greatly increases the risk of false positives. Barring strong specific a-priori hypotheses, this risk must be mitigated by multiple comparison corrections (or statistical parametric mapping in the case of multiple electrodes). Studies published before 2000 often cast a wide net without mentioning these methods. As the next section shows, however, the findings are surprisingly consistent across studies, suggesting that few of the reported positives were false.

#### Research findings

**Figure 2:**
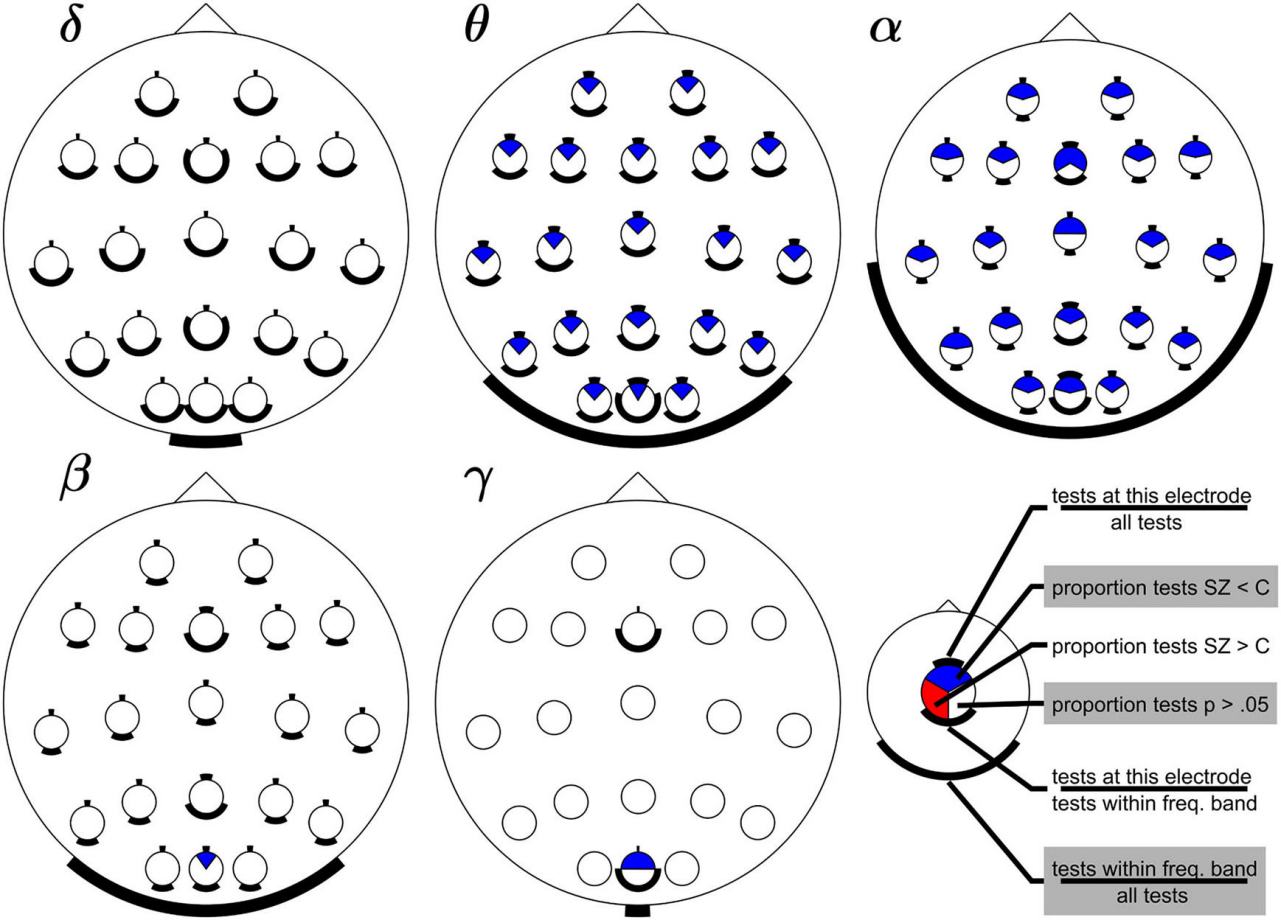
Summary of changes in SSVEPs in Sz compared to controls across electrodes and frequency bands. Subplots show results for SSVEPs that fall within the delta, theta, alpha, beta, and gamma frequency bands (Methods). Each circle within a head represents an electrode. Blue areas indicate the proportion of significant reductions in SSVEPs in Sz compared to healthy controls. Red areas indicate significant increases (none were reported), and white areas indicate the proportion of non-significant tests. The black bars help interpret these proportions relative to the number of tests performed at each electrode and in each band. The bar on top of each electrode indicates the proportion of tests at that electrode and within that frequency band compared to all 228 tests included in our analysis. The bar at the bottom of each circle indicates the proportion of tests that were performed at that electrode but now relative to the number of tests across electrodes within that frequency band. The bar at the bottom of each head indicates the proportion of tests within that frequency band (across all electrodes). The absence of red shows that no significant increases in SSVEPs in Sz compared to controls were reported. The preponderance of blue shows that SSVEPs in Sz are often smaller than in controls across a wide range of electrode locations and across all frequency bands, except the delta band.

Figure 2 summarizes the results of the 228 tests (across 15 studies) comparing the strength of the SSVEPs in people living with schizophrenia and controls (Methods). The most striking feature is that compared to controls, people with schizophrenia had widespread reductions (blue) in SSVEPs across electrodes and stimulus frequencies; not a single test across these studies reported a significant increase in SSVEPs (red). Under the null hypothesis of no effect, one would expect approximately equal red and blue areas. Hence, this graph supports the claim that SSVEPs are reduced in schizophrenia.

We note that the large fraction of non-significant results (white) only shows the absence of evidence, not evidence of the absence of an effect. The relative size of the blue and white area is related to the effect size: across studies with equal power, the fraction of significant findings increases with effect size. With some simplifying assumptions (Methods), we can invert this relationship to provide a coarse estimate of the Cohen's D effect size, based on the fraction of significant tests. We calculated this for each electrode and band with evidence to support an effect (i.e., those with blue areas in Figure 2) and found that the mean effect size was 0.48 with little variation across bands/electrodes (standard deviation 0.08). In other words, the relative size of the white and blue areas in Figure 2 corresponds to a medium size effect.

The evidence in favor of decreases is especially strong for stimulus frequencies within the theta and alpha band, which have been targeted by multiple studies and with multiple electrodes locations. In contrast, stimulus frequencies in the gamma band have only been evaluated at electrode sites Oz and Fz and only in a single study (Krishnan et al., 2005). The circular bars below the topographic plots reflect this bias in the sampling of frequency bands, and the circular bars below each electrode reflect the bias in the sampling of electrode positions. This visualization emphasizes that the strength of evidence should be evaluated with respect to the number of relevant studies to avoid interpreting the absence of evidence as evidence of absence.

Hence, even though the evidence of reduced SSVEPs is clearly strongest for frequencies within the theta and alpha bands, there are several reasons why this may not reflect a true frequency-dependent difference between Sz and controls. First, sampling across bands is uneven (only 1 study evaluated power in the gamma band; Krishnan et al., 2005). Second, SSVEPs tend to be weaker for higher stimulus frequencies (Herrmann, 2001; Pastor et al., 2003). As a result, a difference in power between groups may be more difficult to detect. Third, SSVEPs at frequencies located in the beta band were almost exclusively quantified with the higher harmonics of lower frequency stimuli (Rice et al., 1989; Jin et al., 1990; Wada et al., 1994) instead of by comparing responses at the first harmonic of a stimulus (Krishnan et al., 2005). These approaches do not necessarily quantify the same underlying mechanisms (see above).

## Discussion

Our review of the literature supports the following qualitative conclusions. First, SSVEPs are never significantly increased in Sz compared to controls–this finding is surprisingly consistent across electrode locations, frequency bands, and studies with widely different experimental approaches. Second, significantly decreased SSVEPs are found in all frequency bands, except the delta band. This finding of decreased SSVEPs, however, needs to be qualified. While decreases in the theta and alpha bands are reported at all electrode locations, decreases in the beta and gamma band have only been found for occipital electrodes. Moreover, the number of studies varies considerably across frequency bands and electrodes, hence the strength of this evidence varies across bands and electrodes. Finally, the large fraction of non-significant tests (white in Figure 2) even for bands in which significant decreases (blue) are common, serve as a reminder that decreases are not found under all conditions and depend on the patterns used to induce SSVEPs (e.g., contrast, color, size, or spatial pattern) (Butler et al., 2001, 2005; Clementz et al., 2004; Kim et al., 2005).

We used the common nomenclature (delta, theta, alpha, beta, gamma) only for convenience–to group measurements of visually driven responses (Methods). Often, however, these bands are used to refer to intrinsic neuronal oscillations. It is important to note that the data reviewed here do not necessarily have any direct bearing on such intrinsic oscillations. In other words, evidence of decreased SSVEPs in Sz does not necessarily imply that intrinsic oscillations in those same bands are reduced in Sz. That said, there are notable changes in intrinsic oscillations in Sz (for review, see Uhlhaas et al., 2008). Uhlhaas et al. (2008) have proposed that these are useful biomarkers for diagnosis and may be linked with specific aspects of cognition that are impaired in Sz [e.g., learning (theta), or attention (alpha, gamma)]. We believe much can be learned from studying driven and intrinsic responses together. Under some circumstances these responses rely on largely non-overlapping cortical circuitry (Zhigalov and Jensen, 2020), while in the alpha band it appears possible to use visual drive to entrain intrinsic responses (Notbohm et al., 2016). A better understanding of these interactions might lead to therapeutic applications in which rhythmic visual drive boosts the intrinsic oscillations that are impaired in Sz (Uhlhaas et al., 2008).

Most studies have analyzed the amplitude of the trial-averaged SSVEPs–the evoked response. Several factors can contribute to reductions in the evoked response. For instance, a reduced coherence in the brain can result in a reduced amplitude measured on the scalp. Similarly, higher phase variability across trials (or, equivalently, reduced intertrial coherence) contributes to a reduced trial-averaged amplitude. Methods that distinghuish between induced and total power (Mast and Victor, 1991; Roach and Mathalon, 2008), or analyze (intertrial) coherence may shed some light on these contributing factors and, by inference, the changes in the underlying circuitry. Currently, no published SSVEP studies in Sz have combined these methods; using them to complement the analysis of evoked responses is a promising direction for future research.

In principle, animal models of Sz provide access to more local and sensitive measures of neural activity (local field potentials as well as spiking activity) and offer a range of possibilities for causal manipulation using pharmacological or optogenetic approaches. In the auditory domain this has led to productive research programs studying auditory evoked potentials (Vohs et al., 2012; Nakao and Nakazawa, 2014; Kim et al., 2015; Shahriari et al., 2016; Sivarao et al., 2016; Wang et al., 2020) but we found no reports investigating visual evoked potentials in animal models of Sz. We recently showed that a subanesthetic dose of the NMDA antagonist ketamine given to a nonhuman primate (NHP) recapitulates specific visual disturbances found in Sz (Schielke and Krekelberg, 2021). Given the similarity of visual processing in NHP and humans (Orban et al., 2004), and the potential translational value of a matched approach in animals and humans (Konoike et al., 2022), we are currently using this NHP model to investigate changes in rhythmic neuronal responses associated with NMDA hypofunction.

A diagnostic marker benefits greatly from simplicity in the experimental design and analysis. However, such simplicity can limit the inferential value of an outcome and thereby the potential advance in our understanding of changes in the underlying circuitry. For instance, if a baseline-corrected signal-to-noise ratio reliably distinguishes between patients and controls, then that is a potentially important finding. However, even if this were the case, we advocate for extensive reporting that includes separate baseline, signal, and noise measures that may provide more insight into the underlying mechanisms. Open-access data sharing could also entice other researchers to analyze a published data set with novel methods. Given that many avenues for analysis remain largely unexplored in this field (see above), this is likely to fill in gaps in our knowledge. Platforms to streamline such efforts include Nemar and OpenNeuro, or general purpose scientific data sharing at OSF.

Experimental designs and analysis methods differ widely across the studies we reviewed. Based on these we made several suggestions for best practices, but it is notable that the outcomes are surprisingly consistent across studies. Therefore, the evidence is strong that SSVEPs are reduced in people living with schizophrenia; that this affects a wide range of stimulus frequencies; and these reductions are often detectable across the entire scalp. The main weaknesses we identified in this subfield are best described as missed opportunities. First, the gamma band has received little attention in the SSVEP literature even though it appears to be a key player in other aspects of visual processing in Sz (Green et al., 2003; Spencer et al., 2003, 2008; Wynn et al., 2005; Riečanskỳ et al., 2010; Grützner et al., 2013). Second, SSVEP analyses often focus only on the evoked response; coherence or induced power measures may provide additional insight. Third, SSVEP studies in animal models are lacking but are necessary to study the underlying mechanisms.

## Data availability statement

The MATLAB (RRID:SCR_001622) code and data are available at the Open Science Framework (RRID:SCR_003238) (https://osf.io/rcaue/). Further inquiries can be directed to the corresponding author.

## Author contributions

AS and BK contributed to conception and design of the review and analyses. AS performed the database search, created the figures and wrote the first draft of the manuscript. BK edited the manuscript, and supervised this work. Both authors contributed to manuscript revision, read, and approved the submitted version.

## Funding

This study was supported by the National Eye Institute (R01EY032744). The funders had no role in study design, data collection and analysis, decision to publish, or preparation of the manuscript.

## Conflict of interest

The authors declare that the research was conducted in the absence of any commercial or financial relationships that could be construed as a potential conflict of interest.

## Publisher's note

All claims expressed in this article are solely those of the authors and do not necessarily represent those of their affiliated organizations, or those of the publisher, the editors and the reviewers. Any product that may be evaluated in this article, or claim that may be made by its manufacturer, is not guaranteed or endorsed by the publisher.

## Author disclaimer

The content is solely the responsibility of the authors and does not necessarily represent the official views of the National Institutes of Health.
